# Contact tracing efficiency, transmission heterogeneity, and accelerating COVID-19 epidemics

**DOI:** 10.1371/journal.pcbi.1009122

**Published:** 2021-06-17

**Authors:** Billy J. Gardner, A. Marm Kilpatrick

**Affiliations:** Department of Ecology and Evolutionary Biology, University of California, Santa Cruz, California, United States of America; London School of Hygiene & Tropical Medicine, UNITED KINGDOM

## Abstract

Simultaneously controlling COVID-19 epidemics and limiting economic and societal impacts presents a difficult challenge, especially with limited public health budgets. Testing, contact tracing, and isolating/quarantining is a key strategy that has been used to reduce transmission of SARS-CoV-2, the virus that causes COVID-19 and other pathogens. However, manual contact tracing is a time-consuming process and as case numbers increase a smaller fraction of cases’ contacts can be traced, leading to additional virus spread. Delays between symptom onset and being tested (and receiving results), and a low fraction of symptomatic cases being tested and traced can also reduce the impact of contact tracing on transmission. We examined the relationship between increasing cases and delays and the pathogen reproductive number R_t_, and the implications for infection dynamics using deterministic and stochastic compartmental models of SARS-CoV-2. We found that R_t_ increased sigmoidally with the number of cases due to decreasing contact tracing efficacy. This relationship results in accelerating epidemics because R_t_ initially increases, rather than declines, as infections increase. Shifting contact tracers from locations with high and low case burdens relative to capacity to locations with intermediate case burdens maximizes their impact in reducing R_t_ (but minimizing total infections may be more complicated). Contact tracing efficacy decreased sharply with increasing delays between symptom onset and tracing and with lower fraction of symptomatic infections being tested. Finally, testing and tracing reductions in R_t_ can sometimes greatly delay epidemics due to the highly heterogeneous transmission dynamics of SARS-CoV-2. These results demonstrate the importance of having an expandable or mobile team of contact tracers that can be used to control surges in cases. They also highlight the synergistic value of high capacity, easy access testing and rapid turn-around of testing results, and outreach efforts to encourage symptomatic cases to be tested immediately after symptom onset.

## Author summary

Contact tracing is a key tool in the control of infectious diseases. However, to successfully contact and quarantine individuals with traditional methods requires time and is limited by available capacity. As cases rise, limited capacity results in only a fraction of contacts being reached each day before the next set of cases is detected. Here we examine the relationships between increasing case numbers, contact tracing efficiency, and the pathogen reproductive number R_t_ (the number of cases infected by each case) and how these relationships vary with delays between symptom onset and tracing initiation, and incomplete participation in the testing and tracing process. We found that under conditions found throughout much of the world, where only 20–40% of symptomatic cases are detected, contact tracing can reduce R_t_ by a maximum of 20%, and this benefit quickly dissolves as contacts needing tracing exceed capacity. Increases in the fraction of symptomatic people being tested and reductions in the time between symptom onset and initiating tracing substantially increase the potential impact of tracing in reducing R_t_ to 60%, but this benefit is also lost as cases rise and contacts needing tracing exceed capacity to do so. Maintaining excess contact tracing capacity and the ability to shift it to areas most in need can substantially reduce pathogen transmission and limit epidemics.

## Introduction

Severe acute respiratory syndrome coronavirus 2 (SARS-CoV-2) emerged in late 2019, spread globally in early 2020, and resulted in rapidly growing local epidemics, large scale mortality, and strains on hospital capacity in many countries [1–4]. Initial outbreaks in most countries were limited only by severe control measures including closing all but essential businesses as well as schools, churches, and other organizations [5,6]. Only a few countries were able to limit transmission with less disruptive public health measures [7–9]. The combination of severe disease control measures and the disease itself have had devastating impacts on economies and societies [10,11]. Following control with strict lockdown measures, most countries attempted to re-open as many business sectors and activities as possible while avoiding a rapid rise in infections.

Although self-isolation of symptomatic individuals, social distancing, and mask wearing have reduced the transmission of SARS-CoV-2, additional interventions, including business closures and working from home, have often been required to keep the pathogen reproductive number R_t_ below 1 [10,12,13]. One public health strategy that has been used to reduce transmission in some countries is testing symptomatic individuals, tracing their contacts to people they may have infected, isolating infected individuals, and quarantining people that may have become infected but have yet to show symptoms or test positive for the virus (hereafter abbreviated TTIS) [8,13,14]. If contacts of cases can be found and quarantined or isolated before or during their infectious period, this can limit onward spread of the virus [15].

Numerous studies have examined the effectiveness and limitations of TTIS on transmission of SARS-CoV-2 and other pathogens [14–27]. Many studies have shown that TTIS can substantially reduce the pathogen reproductive number, R_t_, but its efficacy depends on the importance of pre-symptomatic and asymptomatic transmission, delays between symptom onset and being tested, and the fraction of infections that are tested and traced [14,19,22,24]. Previous studies have explored various parameter values for contact tracing efficacy by varying the fraction isolated, the fraction symptomatic, and the contribution to transmission of undetected infections [14,19,22]. A key unexplored challenge in implementing TTIS is that tracing contacts and ensuring they can safely quarantine or isolate is a time-consuming process which results in only a fraction of contacts being reached if cases and their contacts exceed case investigation and contact tracing capacity. This reduces the effectiveness of contact tracing as cases increase. Previous studies have assumed fixed values for contact tracing parameters, or have simulated epidemics with models that do not describe the links between cases and their contacts, or use models that don’t include the interactions between rising cases, delays between symptom onset and tracing, and reductions in the pathogen reproductive number R_t_.

Our aim was to examine the relationship between increasing cases, contact tracing efficacy, and the pathogen reproductive number, R_t_, and to examine the potential outcomes for disease dynamics. We built a compartment model of SARS-CoV-2 transmission, parameterized it with data from the literature, and examined how R_t_ varied with number cases traced, delays between symptom onset and the start of contact tracing, the numbers of contacts per case, and different fractions of symptomatic cases being tested and traced. We also simulated a stochastic version of the model with variable numbers of initial infections and with and without contact tracing to examine how reductions in R_t_ and initial conditions affected variation in the timing of epidemics.

## Methods

We built a susceptible-exposed-infected-recovered (SEIR) compartment model of SARS-CoV-2 that included two compartments for infected individuals that reflect the presence of symptoms (pre-symptomatic, *I*_*ps*_, and symptomatic, *I*_*s*_; S1 Fig). For simplicity, we omitted asymptomatically infected individuals (i.e. those that never develop any symptoms) because meta-analyses [28,29], as well as subsequent studies [30], showed that asymptomatic individuals infect a much smaller fraction of their contacts than individuals that eventually develop symptoms. We also omitted severely symptomatic individuals because they are likely to be hospitalized and thus cause very few infections in the community. In addition, contact tracing severely symptomatic cases is ineffective because, by the time an infection progresses to severe symptoms 6–8 days after symptom onset [31,32], many of their contacts will already have finished most of their infectious period, and quarantining or isolating these contacts would have little effect. We note that models that included both of these classes of infected hosts produced results that were very similar to those described below. We also omitted more complex population structure (e.g. household clustering) in order to develop a more transparent relationship between rising cases, delays, and contact tracing efficacy.

The equations of the model (S1 Fig) are:

$$\begin{array}{l} dS/dt=-\kappa \beta S/N(\sigma_{Ips}I_{ps}+\sigma_{Is}I_{s})\\ dE/dt=\kappa \beta S/N(\sigma_{Ips}I_{ps}+\sigma_{Is}I_{s})‐(q_{E\to Ips}+f_{tr}\varepsilon_{E\leftarrow Ips}+f_{tr}\varepsilon_{E\leftarrow Is})E\\ dI_{ps}/dt=q_{E\to Ips}E‐(q_{Ips\to Is}+f_{tr}\varepsilon_{Ips\leftarrow Ips}+f_{tr}\varepsilon_{Ips\leftarrow Is})I_{ps}\\ dI_{s}/dt=q_{Ips\to Is}I_{ps}‐(\tau_{Is}+\gamma_{Is}+f_{tr}\varepsilon_{Is\leftarrow Ips}+f_{tr}\varepsilon_{Is\leftarrow Is+}\alpha_{Is})I_{s}\\ dQ/dt=f_{tr}(\varepsilon_{E\leftarrow Ips}+\varepsilon_{E\leftarrow Is})E+f_{tr}(\varepsilon_{Ips\leftarrow Ips}+\varepsilon_{Ips\leftarrow Is})I_{ps}+(\tau_{Is}+f_{tr}(\varepsilon_{Is\leftarrow Ips}+\varepsilon_{Is\leftarrow Is}))I_{s}‐\gamma_{Q}Q‐\alpha_{Q}Q\\ dR/dt=\gamma_{s}I_{s}+\gamma_{Q}Q \end{array}$$ [Eq 1]

Parameter values are given in Table 1. κ is a social distancing factor between 0 and 1 that scales the transmission rate β (and directly scales the reproductive rate R_t_). σ is the relative infectiousness for the two I classes. q are the transition rates between classes given by the subscripts separated by the arrow (q_E→Ips_ is the transition rate between the E and I_ps_ classes). τ_Is_ is the rate of testing and removal of symptomatic infected individuals I_s_. The terms f_tr_*ε are the removal rate of individuals by contact tracing, with the ε values being the maximum rate of removal by contact tracing from the first subscripted class, due to infections that were caused by the second subscript (e.g. ε_E←Ips_ is the rate that individuals in the E class are removed by contact tracing that were infected by I_ps_ individuals), and f_tr_ is the fraction between 0 and 1 that are actually removed, due to limited contact tracing capacity (see below). Q is the quarantine/isolation class, α are the disease-caused death rates, and γ is the recovery rate to the R class.

**Table 1:** Parameter values and descriptions. Time units for each parameter are either time in days (d), the inverse of time (d^-1^) or unitless (-).

Para-meter	Value/range	Unit	Description	Reference or Derivation
κ	0–1	-	Social distancing factor	Adjusted to produce R_t_ ≅ 1.2–1.7; consistent with data post-lockdown; [56]
β	0.35	d^-1^	Transmission rate	Set to give plausible pre-lockdown R_0_ ≅ 3 [56]
σ_ps_	1.81	-	Relative infectiousness pre-symptomatic:mildly symptomatic	[52]
σ_Is_	1	-	Relative infectiousness	(reference level)
q_E→Ips_	1/3.2	d^-1^	1/(duration latent period)	[52]
f_tr_	Eq 2	-	Fraction of infected individuals traced	See Eq 2; ratio of contacts needing tracing to tracing capacity
N_CT_	15	-	Number of contact tracers for pop of 100,000 people	https://www.naccho.org/uploads/full-width-images/Contact-Tracing-Statement-4-16-2020.pdf
N_CCTD_	12	d^-1^	Number of contacts reached per contact tracer per day	One contact reached each 40 min
N_cpc_	5, 10, 20, 30	-	Number of contacts per case	[39,57]
ε_x←y_	Eq 7	d^-1^	6 maximal contact tracing removal rates; one for each infected class x infected by infected class y	See Eq 7; product of: fraction of symptomatic cases detected by testing; fraction of individuals infected by I_ps_ or I_s_; and fraction of infected contacts still in that infectious class
q_Ips→Is_	1/2.3	d^-1^	1/(duration pre-symptomatic period)	[52]
τ_Is_	0.1–1	d^-1^	Testing removal rate for I_s_ (1/delay from onset to testing & tracing)	Scenarios explored
γ_Is_	1/5,	d^-1^	Recovery rate	[52]
α_Is_, α_Q_	0.0025	d^-1^	Disease caused death rates	estimated using infection fatality ratio (IFR), adjusted for excluding asymptomatics; IFR = 0.0066/0.8; [58–60]; α_Q_ = q_Q→R_*IFR/(1-IFR); α_Is_ = **γ**_Is_*IFR/(1-IFR)
γ_Q_	1/14	d^-1^	Quarantined recovery rate	Does not affect dynamics
f_te_	Eq 3	-	Fraction of symptomatic cases that are tested before they recover	Eq 3; ratio of testing rate to sum of testing rate and recovery rate
f_Ips_, f_Is_	Eq 4	-	Fraction of infected individuals that were infected by pre-symptomatic or symptomatic individuals	Eq 4; relative infectiousness multiplied by infectious period divided by sum of all infections
δ_Ips_, δ_Is_	Eq 5	d	Delay from infection until being traced	Eq 5; the sum of testing delays, time for tracing (0.5d) and for δ_Ips_ the time between being infected by pre-symptomatic individuals and those individuals being detected by testing as symptomatic cases
f_x←y_	Eq 6	-	Fraction of infected contacts infected by class y still in infected class x	Eq 6; Exponentially decaying fraction of individuals that have reached a given class x and still remain there after a given delay δ

The fraction of contacts that can be reached and placed in quarantine each day, f_tr_, is simply the contact tracing capacity (the number of contact tracers, N_CT_ multiplied by the number of calls they can make per day N_CCTD_), divided by the number of contacts that need to be reached each day (the number of symptomatic cases detected by testing, I_s_τ_Is_ multiplied by the number of close contacts per case, N_cpc_):

$$f_{tr}=N_{CT}N_{CCTD}/I_{s}\tau_{Is}N_{cpc}$$ [Eq 2]

If tracing capacity exceeds contacts requiring tracing, all contacts are traced, so max f_tr_ = 1. We note that contact tracing includes both case investigation to obtain information on contacts, and successfully reaching contacts and ensuring they can safely quarantine or isolate themselves. The capacity of a given public health jurisdiction reflects whichever part of this process is limiting.

The maximum contact tracing removal rates ε are derived from the fact that all infected individuals in the E, I_ps_, and I_s_ classes were infected by individuals in the I_ps_ and I_s_ classes, at some time in the past. The maximum removal rates ε are the product of three terms:

1. the fraction of symptomatic cases I_s_ that are tested, f_te_, before they recover:
$$f_{te}=\tau_{Is}/(\tau_{Is}+\gamma_{Is}),$$ [Eq 3]
2. the fraction of individuals in a given class that were infected by pre-symptomatic, I_ps_, or symptomatic, I_s_ individuals,
$$\begin{array}{l} f_{Ips}=(\sigma_{Ips}/q_{Ips\to Is})/[\sigma_{Ips}/q_{Ips\to Is}+\sigma_{Is}/(\tau_{Is}+\gamma_{Is})]\\ f_{Is}=[\sigma_{Is}/(\tau_{Is}+\gamma_{Is})]/[\sigma_{Ips}/q_{Ips\to Is}+\sigma_{Is}/(\tau_{Is}+\gamma_{Is})], \end{array}$$ [Eq 4]
  and
3. the fraction of individuals that are still in infected class *x* (E, I_ps_, or I_s_) by the time they are traced (S2 Fig), f_x←y_ = e^-λδ^, where y is the class they were infected by (I_ps_ or I_s_), λ is the average rate that individuals leave a class (or a series of host classes), and δ is the delay from infection until being traced, which is the sum of the average time to test and trace individuals after symptom onset, 1/τ_Is_, the average time to trace contacts (0.5 days, since each day a new set of contacts arise), and, for infections caused by I_ps_ individuals, the delay between I_ps_ individuals infecting the contacts, and when I_ps_ individuals are identified by testing in the I_s_ class, 1/q_Ips→Is_:
$$\begin{array}{l} \delta_{Ips}=1/\tau_{Is}+0.5+1/q_{Ips\to Is}\\ \delta_{Is}=1/\tau_{Is}+0.5 \end{array}$$ [Eq 5]
  The fraction remaining in each of the 3 infected classes, E, I_ps_, I_s_, that were infected by each of the two infectious classes, I_ps_ and I_s_, respectively, is thus given by six equations:
$$\begin{array}{l} f_{E\leftarrow Ips}=e^{‐(qE\to Ips*\delta Ips)}\\ f_{E\leftarrow Is}=e^{‐(qE\to Ips*\delta Is)}\\ f_{Ips\leftarrow Ips}=(1‐f_{E\leftarrow Ips})(e^{‐(\delta Ips/(1/qE\to Ips+1/qIps\to Is)})\\ f_{Ips\leftarrow Is}=(1‐f_{E\leftarrow Is})(e^{‐(\delta Is/(1/qE\to Ips+1/qIps\to Is)})\\ f_{Is\leftarrow Ips}=(1‐f_{E\leftarrow Ips}‐f_{Ips\leftarrow Ips})(e^{‐(\delta Ips/(1/qE\to Ips+1/qIps\to Is+1/\gamma Is)})\\ f_{Is\leftarrow Is}=(1‐f_{E\leftarrow Is}‐f_{Ips\leftarrow Is})(e^{‐(\delta Is/(1/qE\to Ips+1/qIps\to Is+1/\gamma Is)}) \end{array}$$ [Eq 6]
  The fraction of infections remaining in each class for variable delays in case symptom onset to testing positive, τ_Is_, is shown in S2 Fig. The maximum removal rates ε are simply the product of these three quantities:
$$\begin{array}{l} \varepsilon_{E\leftarrow Ips}=f_{te}f_{Ips}f_{E\leftarrow Ips}\\ \varepsilon_{E\leftarrow Is}=f_{te}f_{Is}f_{E\leftarrow Is}\\ \varepsilon_{Ips\leftarrow Ips}=f_{te}f_{Ips}f_{Ips\leftarrow Ips}\\ \varepsilon_{Ips\leftarrow Is}=f_{te}f_{Is}f_{Ips\leftarrow Is}\\ \varepsilon_{Is\leftarrow Ips}=f_{te}f_{Ips}f_{Is\leftarrow Ips}\\ \varepsilon_{Is\leftarrow Is}=f_{te}f_{Is}f_{Is\leftarrow Is} \end{array}$$ [Eq 7]

**Fig 1:**
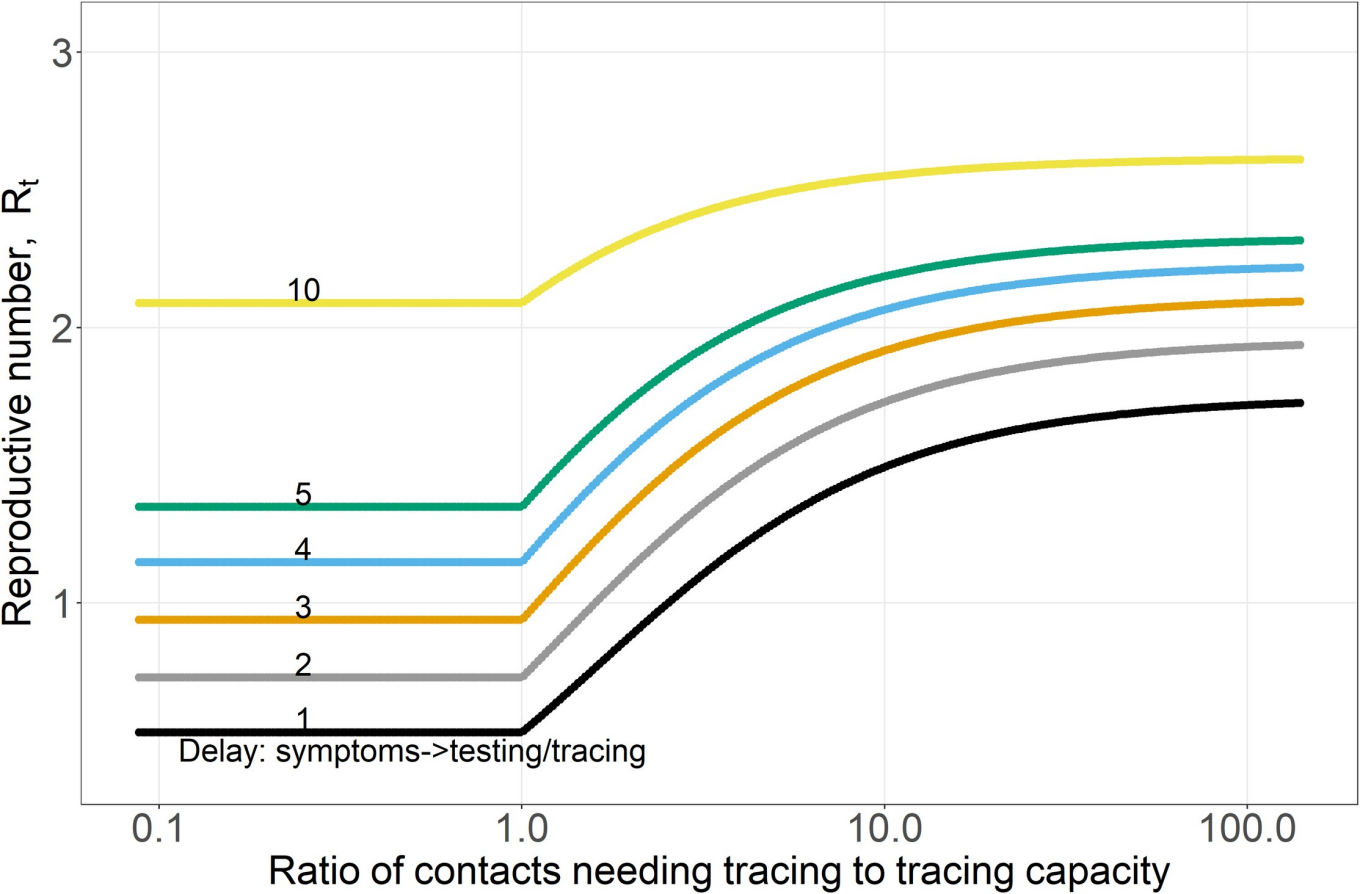
Pathogen reproductive number, R_t_, plotted against the ratio of contacts needing tracing to contact tracing capacity for variable delays (1/τ_Is_) of 1–5 and 10 days between case symptom onset and the start of contact tracing (including getting tested and receiving result). With testing, but no contact tracing, R_t_ increases 35% from 1.7 to 2.3 as the delay 1/τ_Is_ increases from 1 to 5 days, which is evident in the y-axis difference between black and green curves in the upper right of the graph where new case burdens are so high contact tracing is ineffective. The delays (1/τ_Is_) are indicated by the small numbers on each curve in the left of the plot. Curves are horizontal where capacity exceeds contacts needing tracing. The number of contacts per case, N_cpc_, was 10.

**Fig 2:**
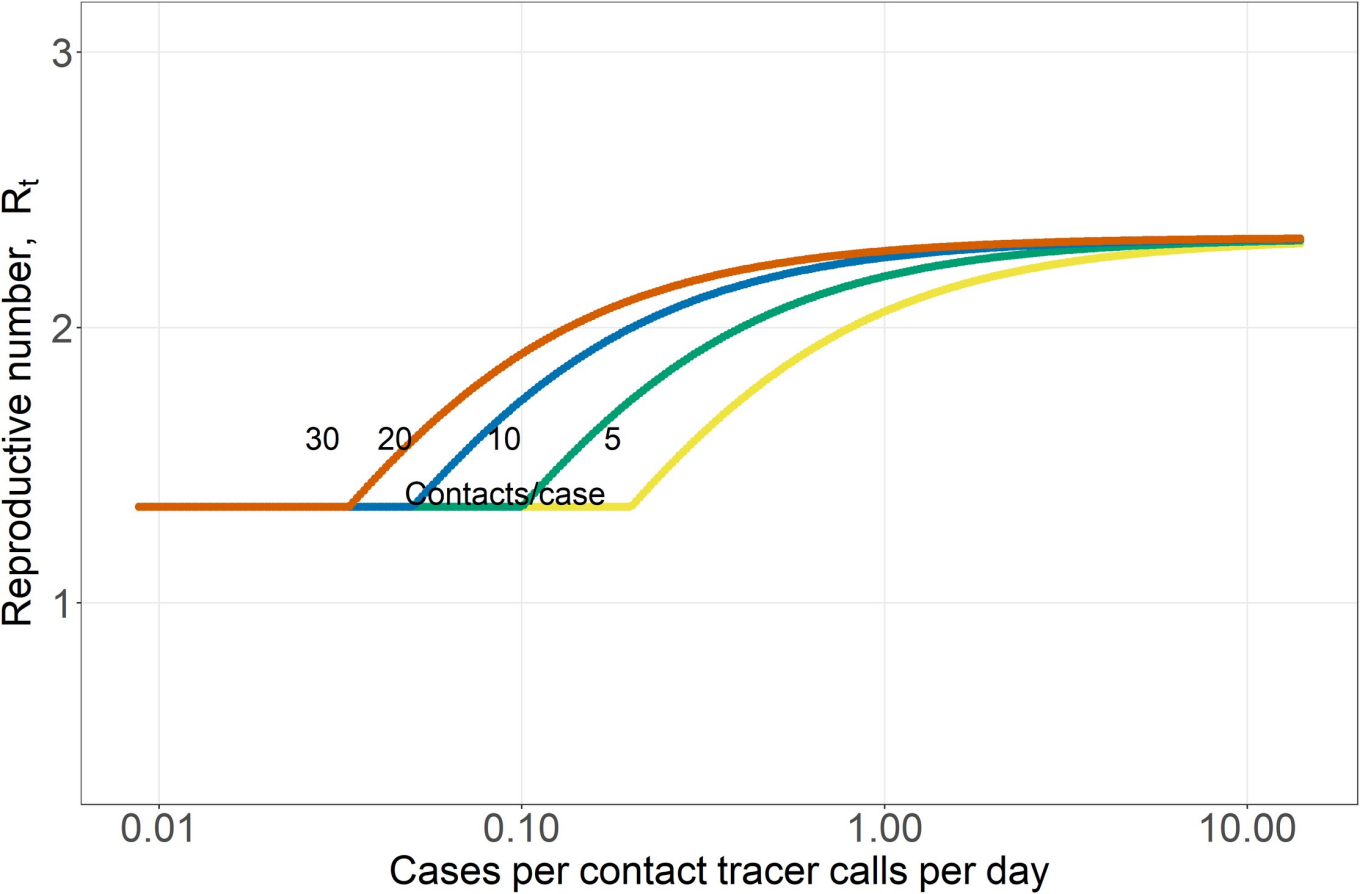
Pathogen reproductive number, R_t_, plotted against the number of cases per contact tracer calls per day, for four different numbers of contacts per case (5, 10, 20, 30; these reflect the range of contacts before and during restrictions on social gatherings [39,45,46]). The number of contacts per case is indicated by the small numbers on each curve in the middle of the plot. The average delay between symptom onset and contact tracing (including getting tested and receiving result), 1/τ_Is_, is set to 5 days; as a result, the green curve is identical to the green curve in Fig 1.

We parameterized the model with data from the literature (Table 1). We note that spatial or temporal variability in contact rates (e.g. from social distancing or other non-pharmaceutical interventions) have direct linear impacts on R_t_ and shift the curves in Figs 1 and 2 vertically (S3 Fig), but do not change their shape and thus don’t affect the proportional impact of contact tracing on R_t_. Increased transmissibility of viral variants such as B.617.2, B.1.1.7 or P.1 [33,34] would similarly shift the curves vertically without changing their shape unless the temporal dynamics of infectiousness are substantially different [35]; at present, data on viral load dynamics for viral variants are sparse [36]. Although the compartmental model above results in exponential distributions for the duration that hosts remain in each host class, the combination of an exposed class E and a pre-symptomatic class I_ps_ results in an incubation period that is approximately lognormally distributed with mean and dispersion similar to the empirically observed values [37]. We explored models with multiple compartments for other classes (e.g. I_s_) to investigate the implications of non-exponentially distributed durations and obtained very similar results to those described below.

We note that while notifying individuals that they have had contact with a case can be done quickly (especially with using a cell-phone tracing app; [22]), successfully ensuring a contact has their needs met (including food, medicine, clothing) to quarantine in a safe space where they won’t infect their household members requires substantially more time (https://www.cdc.gov/coronavirus/2019-ncov/php/notification-of-exposure.html). Thus, we assumed the approximate duration required for a successful contact tracing call was 40 min, resulting in the number of contacts reached by a contact tracer per day, N_CCTD_, of 12.

We derived an expression for the pathogen reproductive number R_t_ for the equations above using the next generation matrix [38]:

$$\begin{array}{l} R_{t}=S/N[\kappa \beta ]*[q_{E\to Ips}/(q_{E\to Ips}+f_{tr}\varepsilon_{E\leftarrow Ips}+f_{tr}\varepsilon_{E\leftarrow Is}]*\\ {}[[\sigma_{Ips}/(q_{Ips\to Is}+f_{tr}\varepsilon_{Ips\leftarrow Ips}+f_{tr}\varepsilon_{Ips\leftarrow Is})]+\\ \left[(\sigma_{Is})/(\gamma_{Is}+\tau_{Is}+f_{tr}\varepsilon_{Is\leftarrow Ips}+f_{tr}\varepsilon_{Is\leftarrow Is}+\alpha_{Is})]*[q_{Ips\to Is}/(q_{Ips\to Is}+f_{tr}\varepsilon_{Ips\leftarrow Ips}+f_{tr}\varepsilon_{Ips\leftarrow Is})]\right] \end{array}$$ [Eq 8]

This expression can be understood as the fraction of the population that is susceptible, S/N, multiplied by the contact rate β (which is scaled by the social distancing factor κ), multiplied by the fraction of hosts that pass through the E class to the I_ps_ class without being removed by contact tracing (1^st^ row), multiplied by the sum of the infections arising from I_ps_ (row 2) and the infections arising from I_s_ (row 3). The infections arising from I_ps_ (row 2) are the product of their infectiousness, σ_Ips_, and the infectious period of the I_ps_ class (the inverse of the losses from the I_ps_ class). The infections arising from I_s_ are the product of their infectiousness, σ_Is_, and the infectious period of the I_s_ class (the inverse of the losses from the I_s_ class) all multiplied by the fraction of hosts in the I_ps_ class that pass into the I_s_ class without being contact traced.

We examined how R_t_ varied with different numbers of new symptomatic cases detected (τ_s_I_*s*_), delays of 1 to 10 days (which captures the range observed during the epidemic) between symptom onset and the start of contact tracing (1/τ_Is_ in Eq 2), and numbers of contacts per case (N_cpc_ in Eq 2) [39]. Rapid tests taken on the day of symptom onset, as used by the United Kingdom starting in April 2021, could result in delays as short as 1 day, whereas the time from symptom onset until test results were returned often exceeded 10 days in the US in 2020 [24,40]. In some figures we used the baseline contact tracing capacity standards suggested by the US National Association of County and City Health Officials (15 contact tracers per 100,000 people; https://www.naccho.org/uploads/full-width-images/Contact-Tracing-Statement-4-16-2020.pdf), but note that because the fraction of contacts traced (Eq 2) is a ratio of four quantities, any combination of values that produce the same number of contacts per contact tracer calls per day will produce the same value of R_t_. Thus, the results are not geographically specific; all that is needed to apply the results to a new setting is the ratio of contacts needing tracing to tracing capacity. We performed a simple sensitivity analysis of this relationship by determining how much R_t_ varied with a ten percent increase or decrease in each model parameter (S3 Fig).

We examined the effect of decreasing contact tracing efficiency as infections increased on disease dynamics and R_t_ by simulating a deterministic version of the model in Eq 1 and plotted R_t_ in real time as an epidemic swept through a population over one year. We note that SARS-CoV-2 epidemics in most countries have consisted of multiple surges or waves [15,41] as restrictions have limited contact rates. However, the relative relationships we show between the reproductive number R_t_ and contact tracing demand relative to capacity are time-insensitive and depend only on infections, contacts and contact tracing capacity, and can be scaled by adjusting the fraction of the population that is susceptible.

Finally, we explored the implications of stochastic variability and contact tracing on infection dynamics in a scenario based on a moderate size city (100,000 people) with partly effective non-pharmaceutical interventions/social distancing (κ = 0.6), resulting in R_t_ = 1.33 with moderately effective testing and contact tracing (equivalent to 1/τ_Is_ = 10 days which results in ~25% of infected individuals being tested or quarantined), or R_t_ = 1.57 without contact tracing. We simulated a stochastic version of the model given by Eq 1 where the number of new infections was drawn from a negative binomial distribution with mean equal to R_t_ and dispersion parameter 0.16 which is intermediate between available estimates for COVID-19 [42–44]. We examined different initial numbers (5 and 50) of latently infected individuals, E, at the start of the epidemic to understand how stochastic variation in transmission could impact the timing of epidemics when the number of initial infections was small or moderately large.

R code to reproduce all results is available from: https://github.com/marmkilpatrick/Contact-Tracing-Efficiency

## Results

The effectiveness of contact tracing in reducing the pathogen reproductive number, R_t_, was dependent on synergistic interactions among three factors: the number of cases being traced (given a fixed number of contact tracers), the delay between symptom onset and the start of tracing, 1/τ_Is_, (including getting tested and receiving result), and the fraction of symptomatic cases that get traced (Figs 1 and 2).

First, the relationship between R_t_ and the number of cases per contact tracer calls per day was approximately sigmoid (Figs 1 and 2); at both high and low case numbers adding or removing contact tracers had smaller effects, whereas at intermediate case numbers relative to capacity, shifting contact tracers had a much larger impact. When the ratio of contacts needing tracing to capacity was high (i.e. 10 or higher), contact tracing had relatively little effect in reducing R_t_ no matter how long the delay was between symptom onset and the start of tracing, 1/τ_Is_ (right side of Fig 1; this pattern is also evident in Fig 2). This is because <10% of the contacts needing tracing and isolation were reached and isolated.

Contact tracers in regions with very high new case numbers relative to contact tracing capacity (>10 on Fig 1) would have a larger reduction on R_t_ if they were tracing calls in locations with intermediate numbers of cases (ratios of contacts needing tracing to capacity of 1–5). In order to reverse an increase in cases (i.e. to reduce R_t_<1), the analyses in Figs 1 and 2 suggest that when new case burdens are high relative to capacity, population-wide interventions (e.g. social distancing or different levels of shelter-in-place or lockdown orders which reduce contact rates, β or κ, and shift the entire curves in Figs 1 and 2 downward proportionately and S3 Fig), or orders of magnitude increases in contact tracing capacity are needed until R_t_ can be effectively reduced by contact tracing. Conversely, and intuitively, when there is excess contact tracing capacity (to the left of 1 on the x-axis in Fig 1), contact tracing was as effective as it could be in reducing R_t_, but excess capacity is underutilized. Shifting a subset of contact tracers to areas where cases and contacts needing tracing exceed capacity could substantially reduce R_t_ in those areas.

Second, increasing delays, 1/τ_Is_, between symptom onset and the start of tracing had a synergistic effect on the efficacy of contact tracing (Fig 1). With a 10 day delay, contact tracing could only reduce R_t_ by 20% (from 2.6 to 2.1 in Fig 1; compare the right and left heights of the yellow curve). In contrast, if all symptomatic individuals got tested within 4 days of symptom onset and results were returned within the next 24 hours (1/τ_Is_ = 5 days), contact tracing could reduce R_t_ by 40% from 2.2 to 1.3 (Fig 1 green curve). The maximum benefit of contact tracing (the difference between the maximum and minimum of each curve) is relatively constant for short delays of 1-3d between symptom onset and testing (1/τ_Is_) because most infected contacts are still in the E class or have just entered the I_ps_ class (S2 Fig). As these delays increase further, more contacts have moved into the infectious classes and have transmitted the pathogen to other hosts (S2 Fig). With a 10 day delay, 96% of contacts have left the E class, and 49% have already reached the R class (S2 Fig). The number of contacts per case obviously also influences the time required to trace these contacts (Fig 2). If allowable (or illegal) gathering sizes increase, this increases the number of contacts per case, which reduces contact tracing efficacy if contact tracing capacity is exceeded.

Thirdly, if contacts for a substantial fraction of all symptomatic cases do not get traced and quarantined, TTIS is far less effective. Figs 1 and 2 primarily showed optimistic scenarios where the fraction of symptomatic infections that are tested and traced is determined only by the delay between symptom onset and testing results being returned (1/τ_Is_) and the rate of recovery. With a 5 day delay (1/τ_Is_ = 5) (Fig 2), this results in 50% of infections being detected by testing in the mildly symptomatic state (I_s_) which is higher than some estimates of case under-ascertainment based on seroprevalence studies, especially early in the pandemic [47,48]. In contrast, if only half of symptomatic cases are tested (and their contacts traced), this is similar to a 10 day delay (1/τ_Is_ = 10) between symptom onset and tracing, which has a far lower impact in reducing R_t_ at both high and low case burdens (the yellow curve in Fig 1). If quarantining contacts is only partly effective (e.g. they stay home but infect household members who go on to infect others) this will similarly reduce the effectiveness of contact tracing.

**Fig 3:**
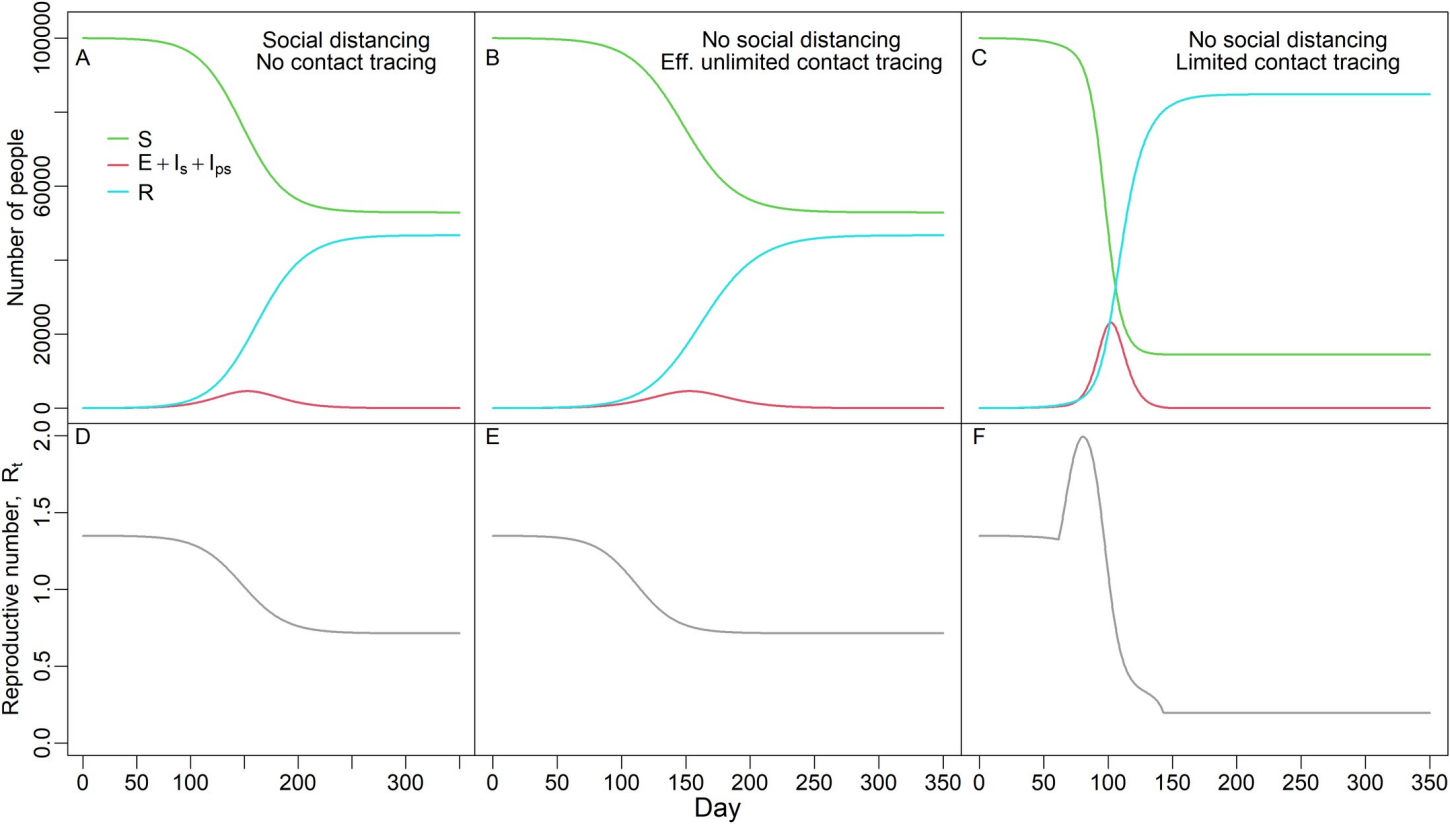
Reduced contact tracing efficiency with increasing cases leads to accelerating epidemics. Top panels (A, B, C) show the number of susceptible, infected (latent, pre-symptomatic and symptomatic combined), and recovered individuals. Bottom panels (D, E, F) show the reproductive number, R_t_, over time. Left most panels (A, D) show dynamics with no contact tracing but social distancing (κ = 0.58) set to give same initial R_0_ (1.35) as with contact tracing. Middle panels (B, E) show dynamics with effectively unlimited contact tracing (1500 contact tracers making 12 calls/day; 10 contacts per case) but no social distancing (κ = 1), with an identical value of R_0_ as in panels A, D. Right panels (C, F) show dynamics with the same parameter values as (B, E) except with limited contact tracing (15 contact tracers). R_0_ is the same value as in panels D and E (R_0_ = 1.35), but R_t_ increases as cases increase and contact tracing becomes inefficient, which overwhelms the decrease in the fraction of the population that is susceptible. In all panels, the delay from symptom onset to receiving test results, 1/τ_Is_, is 5d. All populations start with 100,000 individuals. Note the identical epidemic sizes (final fraction susceptible 0.53) for panels A (social distancing) and B (effectively unlimited contact tracing), but much larger epidemic size for limited contact tracing in panel C (final fraction susceptible 0.14).

Limited contact tracing can also produce unexpected dynamics. Reduced contact tracing efficiency with increasing cases results in a transient accelerating epidemic where R_t_ actually increases over time leading to a much larger epidemic size and an accelerated epidemic (Fig 3E and 3F). A decrease in contact tracing efficiency as cases rise can, initially, outweigh the depletion of susceptible individuals which leads to a spike in R_t_ over time, until depletion of susceptibles overwhelms this effect (compare rightmost panels, Fig 3E and 3F, which have limited contact tracing, to leftmost panels, Fig 3A and 3B, where social distancing reduces R_t_ to the same initial value as contact tracing). With effectively unlimited contact tracing this phenomenon does not arise (compare middle panels, Fig 3C and 3D, which show effectively unlimited contact tracing, to rightmost panels Fig 3E and 3F).

**Fig 4:**
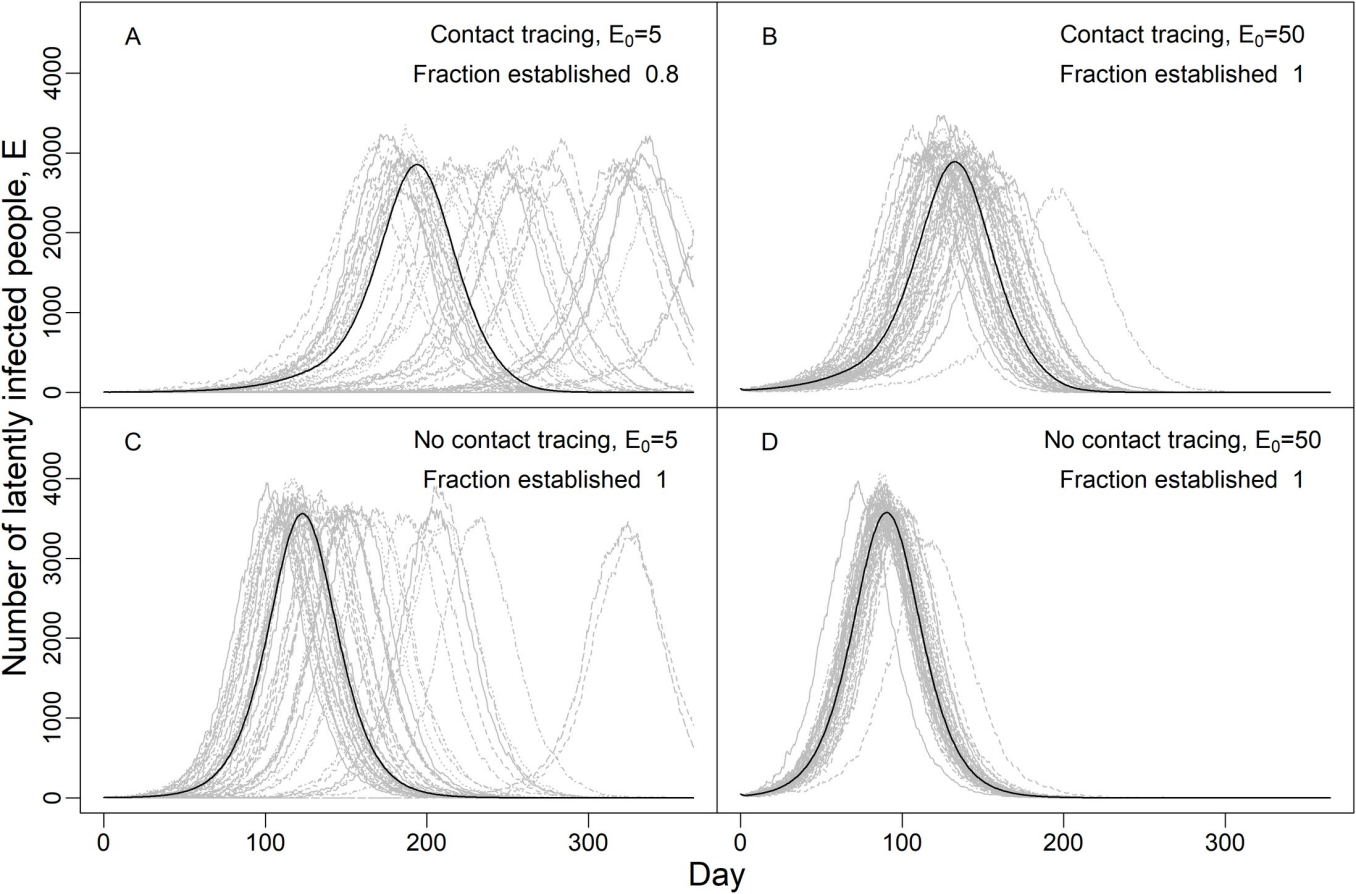
Variability in the timing and outcome of epidemics due to stochastic variation in individual transmission. Lines show number of latently infected individuals in the E class over time for 1 year with moderate social distancing that reduces contact rates by 40% (κ = 0.6). Grey lines show runs from a single stochastic simulation and the black line shows the deterministic outcome. The fraction of epidemics that establish is the fraction of simulations where the maximum number of people infected at any time exceeds the starting number infected. The four scenarios shown include different starting numbers of latently infected individuals on day 0, E_0_ (A, C: 5; B, D: 50), and with (A, B) or without (C, D) contact tracing (CT) which lowered R_0_ from 1.57 to 1.33. The delay from symptom onset to testing and tracing 1/τ_Is_ was 10d. The modeled population of 100,000 people had 15 tracers making 12 calls/day, and each case had an average of 10 contacts which is intermediate between pre-lockdown and lockdown conditions; this scenario is the same as the yellow line in Fig 1.

Finally, the impact of contact tracing in reducing the pathogen reproductive number R_t_ has two consequences on the temporal timing and establishment of epidemics. First, as is well known, reducing R_t_ delays and reduces the peak of the epidemic (Fig 4 top vs bottom panels). Second, and less appreciated during the current pandemic, stochastic variation in R_t_ can lead to very different timing of epidemics if the initial number of infected individuals is low (Fig 4A and 4C; epidemic peaks can vary by 6 months by chance), and variation is larger if R_t_ is lower (Fig 4A vs Fig 4C and Fig 4B vs Fig 4D). Finally, heterogeneity in individual transmission can result in local fadeout of the pathogen and fadeout is more likely when contact tracing reduces R_t_ closer to 1, and if the number of initially infected individuals is lower (compare the Fraction of epidemics established in panels A vs B-D).

## Discussion

The two main strategies that have been used to control SARS-CoV-2 transmission before the development of vaccines were TTIS and society-wide social distancing interventions (including closing businesses, banning gatherings, wearing masks, etc.) [12]. Closing businesses has had devastating impacts on employment and economies, as well as cascading impacts on society. TTIS has far smaller economic and societal costs, but its efficacy in controlling epidemics is not fully understood, and some studies suggest that it is insufficient to keep R_t_ below 1 in many settings, especially without widespread testing and digital contact tracing [13,14,22,49]. We examined how the efficacy of contact tracing decreases with increasing case burden. As case burdens increased relative to contact tracing capacity, contact tracing reached a small fraction of contacts and had little effect on R_t_, leading to accelerating epidemics. Conversely, when case numbers were very low relative to contact tracing capacity, there was excess capacity and, all else being equal, contact tracers could be used more effectively in higher case burden settings with negligible impacts on local transmission. We note that the exact number of contact tracers needed to reduce R_t_ depends on the number of contacts per case and the number of calls each tracer can make each day (Fig 2). However, this key quantity appears as a ratio of case-contacts per contact tracer calls per day. Thus, each contact tracing team (e.g. a county or local jurisdiction) can use local estimates of contacts per case and the number of calls each tracer can make each day to determine where they are on the modelled relationships (Figs 1 and 2).

Allocating contact tracers among populations to most effectively reduce total infections is a non-trivial problem. A smaller reduction in R_t_ (e.g. 10%) in one population can prevent more infections (especially over multiple generations of transmission) than a larger (e.g. 20%) reduction in R_t_ in a second population if R_t_ in the second location is lower (especially when R_t_>1 in the first population), or when there is a larger number of infected individuals in the first population. Thus, transferring contact tracers from a region with a high case burden relative to contact tracing capacity to maximize their efficacy in reducing R_t_ should only be done if other measures (e.g. social distancing) can be put into place to reduce R_t_ where case numbers are high. More generally, allocation of contact tracers to maximize the number of cases prevented given an array of tools would require a complex dynamic analysis beyond that examined here. However, the results shown here show the quantitative impact that shifts in contact tracers can have in reducing R_t_, which can be critical, especially if the goal is to use contact tracing to reduce R_t_<1 in a given population.

We also found that the efficacy of contact tracing itself, regardless of capacity, was strongly influenced by delays between the onset of symptoms and the beginning of tracing, as well as the fraction of symptomatic infections that were traced. Unless delays were short and the fraction of symptomatic cases that were traced was high, contact tracing had limited effects in reducing R_t_. This finding of synergistic effects between testing delays and contact tracing efficiency parallels results from other studies demonstrating the large effects of delays in reducing efficacy of isolating infections by testing alone [50]. We note that in the model considered here, only symptomatic individuals were removed by testing (pre-symptomatic individuals were not detected by testing) which leads to a smaller impact of testing on R_t_ than is possible if all individuals are tested [40]. Our results emphasize the importance of encouraging people to get tested as soon as possible after mild symptom onset, and having sufficient testing capacity to return their results quickly [40]. Similarly, the fraction of symptomatic infections that get tested and traced is poorly known, but if the ratio of infections to cases from seroprevalence studies in some locations is approximately correct (e.g. 10:1 to 4:1; [47,48]), then contact tracing will have limited effects in reducing transmission.

Allocation of contact tracing resources can be most efficiently deployed in two ways. First, contact tracing is much more effective when infections are detected soon after symptom onset. One should prioritize these individual cases for tracing since their contacts are likely to be earlier in their infections and quarantining/isolating them will cut off most or all of their infectious period (S2 Fig). If one knows the date of contact between the case and the contact, one could also prioritize tracing more recent contacts and those that had contact with the case during the case’s days of peak infectiousness just before and after symptom onset [51,52]. Second, if one is attempting to limit transmission in multiple regions (e.g. counties within a state) one could deploy contact tracers to counties where they will be able to have the most impact: from places with excess capacity to those with intermediate numbers of cases per contact tracer calls per day. Conversely, if contact tracers cannot quarantine the contacts of cases within 10–12 days of the case’s symptom onset, they will be unlikely to effectively reduce transmission from those contacts.

Our results also demonstrate two phenomena observed in COVID-19 epidemics. First, epidemic dynamics sometimes differ enormously between places that seem otherwise similar which is well understood by mathematical modelers [15,53–55], but sometimes forgotten in considering spatial variation in epidemic outcomes. Spatial variation in disease dynamics may be due to differences in social behavior or contact patterns, but we showed that stochastic chance may also play a large role in shifting the timing of epidemics by up to six months. The impact of stochasticity is largest when the initial number of cases is low and R_t_ is close to 1 (i.e. when lockdowns are first lifted), because this results in populations spending longer periods of time with few cases where stochastic variation is most important. Second, staged business re-openings and the emergence of new virus variants have sometimes led to accelerating or runaway epidemics. These may be due to sudden changes in social behavior, but we showed that accelerating epidemics can also result from decreases in contact tracing efficiency [26]. Increasing contact tracing capacity could limit this epidemic acceleration as cases increase, which suggests that training a reserve capacity of tracers and being able to deploy a mobile tracing force could help limit runaway epidemics.

More broadly, contact tracing could play an important role in limiting transmission of SARS-CoV-2 and other pathogens [49]. However, we found that its efficacy depends on participation in seeking testing immediately following symptom onset, quick return of test results, and sufficient contact tracing capacity if case numbers surge. Shortcomings in each of these factors greatly limit its efficacy, especially as cases increase, which could necessitate much more damaging measures to control transmission, including widespread business and school closures. Investments in public health, including testing, contact tracing, and public outreach to encourage health seeking when symptomatic, is likely a much more cost-effective approach to control COVID-19, and other diseases.

## Supporting information

S1 Fig Compartmental model of SARS-CoV-2. See text for equations and Table 1 for parameter values. Boxes represent Susceptible (S), Exposed (E), Infected (I), recovered (R), and Quarantined/Isolated (Q) classes. There are two compartments for infected individuals that reflect the presence of symptoms (pre-symptomatic, *I*_*ps*_, and symptomatic, *I*_*s*_). κ is a social distancing factor between 0 and 1 that modifies the contact rate β, σ are infectiousness for each of the I_ps_ and I_s_ classes, q are transition rates between classes given by the subscripts separated by the arrow (e.g. q_E→ps_ is the transition rate between the E and I_ps_ classes), ε are the rates of removal by contact tracing from the E or I classes to the quarantined class Q based on which class infected those individuals (e.g. ε_E←Ips_ is the contact tracing removal rate of E individuals that were infected by I_ps_ individuals), τ_Is_ is the removal rate by testing of symptomatic infected individuals, α is the disease-caused death rate, and γ are the recovery rates to the R class. The dashed lines indicate that both classes of infected individuals contribute to transmission.
(TIF)

S2 Fig The impact of delays between individuals becoming infected and being traced and removed on the host class that that infected individual will be in before being removed. For example, if the delay between infection and quarantine/isolation is 6 days, then 15% of infected individuals will still be in the latently infected class, E, 28% in the pre-symptomatically infected class, I_ps_, 33% in the symptomatically infected class, I_s_, and 24% will have already recovered, R.
(TIF)

S3 Fig Sensitivity analysis. The plot shows how much R_t_ changes from a 10% increase (red) or 10% decrease (blue) in that model parameter relative to values in Table 1 (with τ_Is_ = 0.2 and κ = 1). R_t_ scales linearly with β and κ, whereas transition parameters q_E→Ips_, q_Ips→Is_, the testing rate τ_Is_, and pre-symptomatic infectiousness σ_Ips_ have approximately 50–75% as large an effect as β or κ. The recovery rate, γ_Is_, and symptomatic infectiousness σ_Is_ are less influential, and the death rate α_Is_ has very little effect on R_t_.
(TIF)
