# Supplementary figures and images for: Contact tracing efficiency, transmission heterogeneity, and accelerating COVID-19 epidemics
Source: PLoS Comput Biol. 2021 Jun 17;17(6):e1009122. doi: 10.1371/journal.pcbi.1009122 (PMC8241027; doi:10.1371/journal.pcbi.1009122)

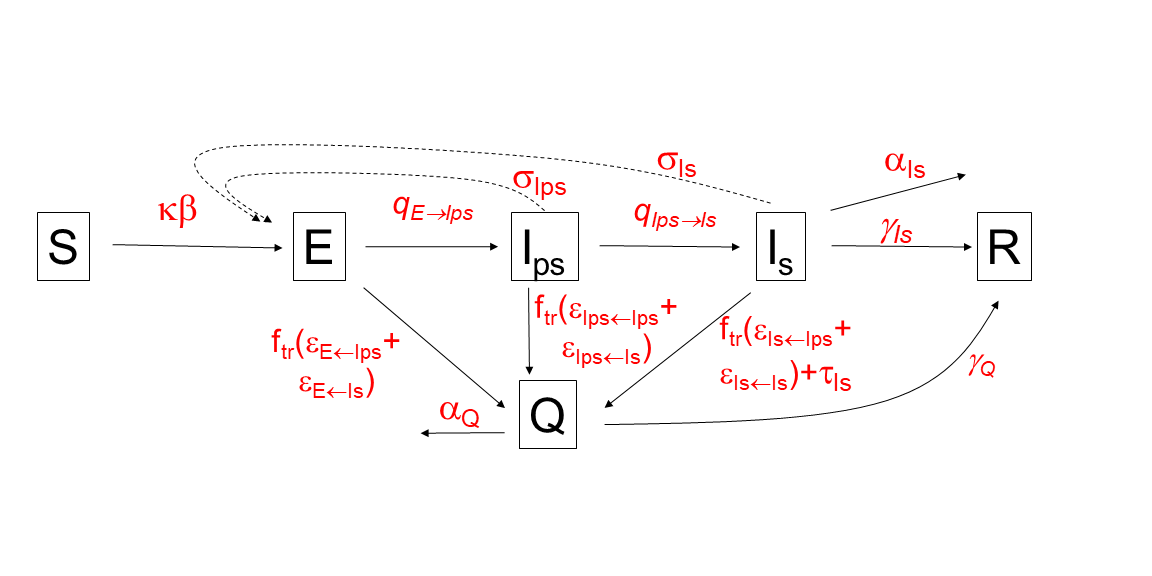

Supplement: S1 Fig — See text for equations and Table 1 for parameter values. Boxes represent Susceptible (S), Exposed (E), Infected (I), recovered (R), and Quarantined/Isolated (Q) classes. There are two compartments for infected individuals that reflect the presence of symptoms (pre-symptomatic, Ips, and symptomatic, Is). κ is a social distancing factor between 0 and 1 that modifies the contact rate β, σ are infectiousness for each of the Ips and Is classes, q are transition rates between classes given by the subscripts separated by the arrow (e.g. qE→ps is the transition rate between the E and Ips classes), ε are the rates of removal by contact tracing from the E or I classes to the quarantined class Q based on which class infected those individuals (e.g. εE←Ips is the contact tracing removal rate of E individuals that were infected by Ips individuals), τIs is the removal rate by testing of symptomatic infected individuals, α is the disease-caused death rate, and γ are the recovery rates to the R class. The dashed lines indicate that both classes of infected individuals contribute to transmission. (TIF) [file pcbi.1009122.s001.tif]

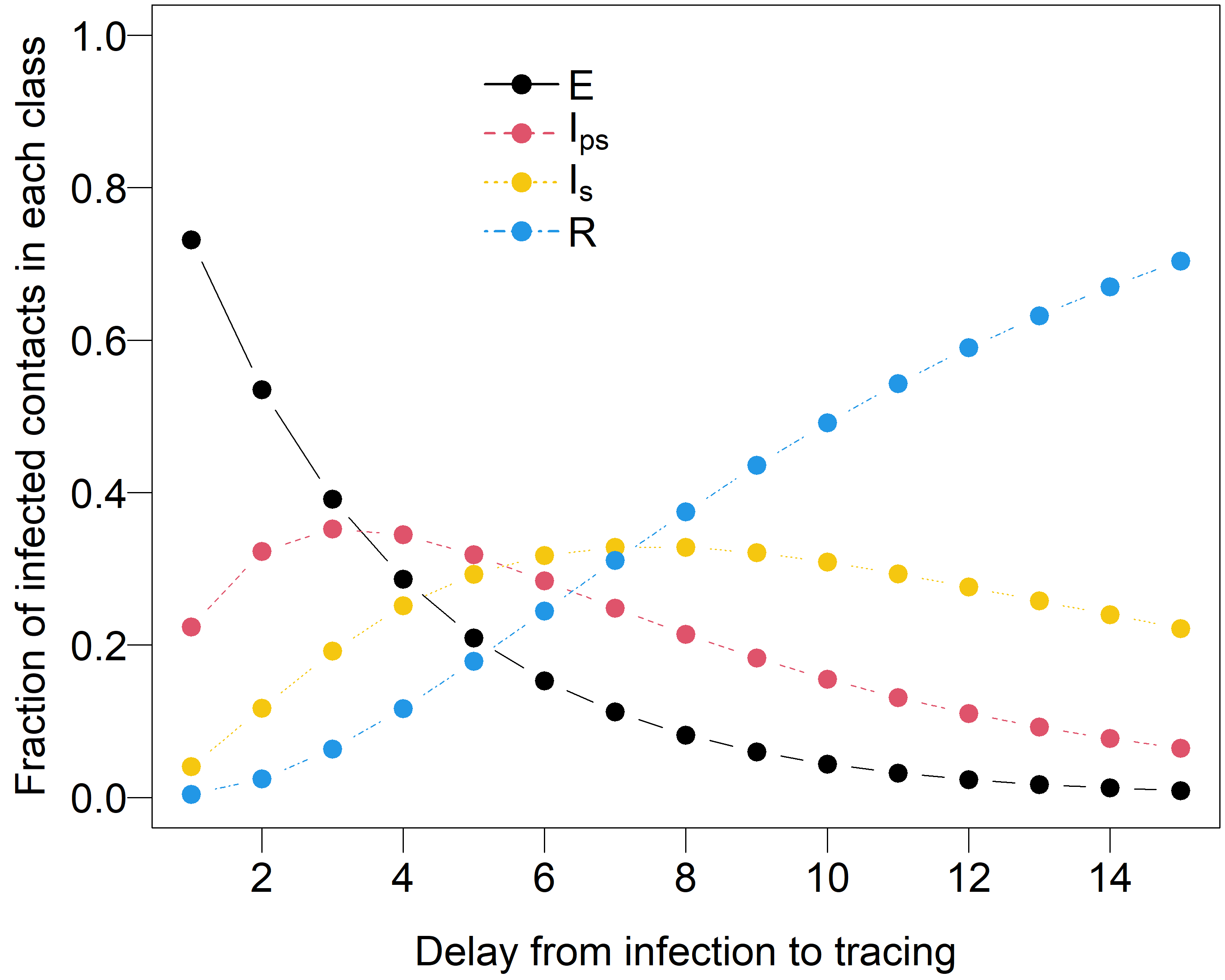

Supplement: S2 Fig — For example, if the delay between infection and quarantine/isolation is 6 days, then 15% of infected individuals will still be in the latently infected class, E, 28% in the pre-symptomatically infected class, Ips, 33% in the symptomatically infected class, Is, and 24% will have already recovered, R. (TIF) [file pcbi.1009122.s002.tif]

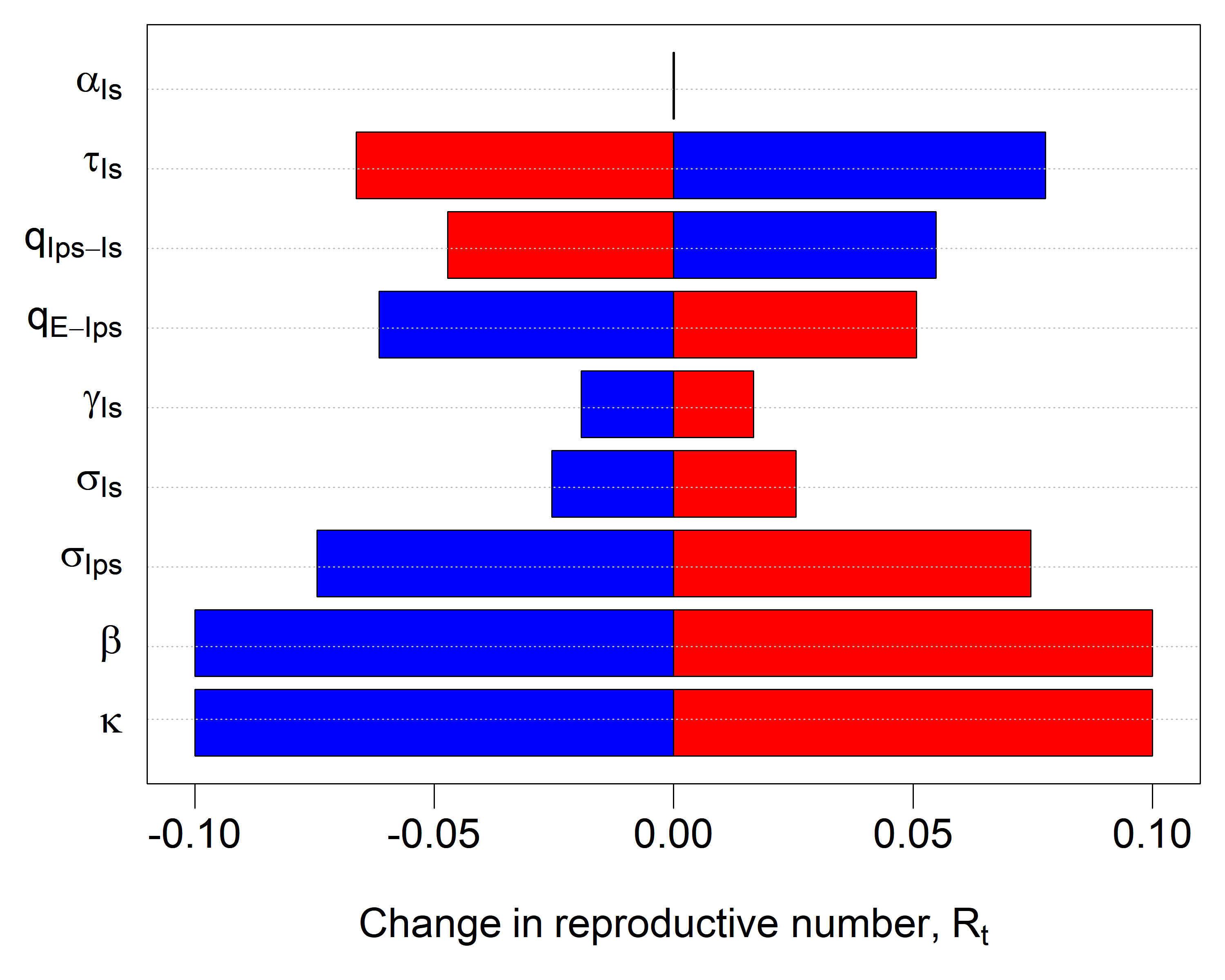

Supplement: S3 Fig — The plot shows how much Rt changes from a 10% increase (red) or 10% decrease (blue) in that model parameter relative to values in Table 1 (with τIs = 0.2 and κ = 1). Rt scales linearly with β and κ, whereas transition parameters qE→Ips, qIps→Is, the testing rate τIs, and pre-symptomatic infectiousness σIps have approximately 50–75% as large an effect as β or κ. The recovery rate, γIs, and symptomatic infectiousness σIs are less influential, and the death rate αIs has very little effect on Rt. (TIF) [file pcbi.1009122.s003.tif]
